# Infant development at 14 months in the context of maternal objective and subjective birth experience and infant hair glucocorticoids

**DOI:** 10.1186/s12887-025-05528-5

**Published:** 2025-03-14

**Authors:** Isabel Jaramillo, Luisa Bergunde, Corinna Müller-Stark, Marlene Karl, Victoria Weise, Clemens Kirschbaum, Susan Garthus-Niegel, Susann Steudte-Schmiedgen

**Affiliations:** 1 Institute and Policlinic of Occupational and Social Medicine, Faculty of Medicine, University Hospital Carl Gustav Carus, TUD Dresden University of Technology, Dresden, Germany; 2 Department of Psychotherapy and Psychosomatic Medicine, Faculty of Medicine, University Hospital Carl Gustav Carus, TUD Dresden University of Technology, Dresden, Germany; 3 Institute of Biological Psychology, Faculty of Psychology, TUD Dresden University of Technology, Dresden, Germany; 4 Institute for Systems Medicine (ISM), Faculty of Medicine, Medical School Hamburg MSH, Hamburg, Germany; 5 Department of Childhood and Families, Norwegian Institute of Public Health, Oslo, Norway

**Keywords:** Birth experience, Birth complications, HPA axis, hair, Cortisol, Cortisone, Infant development, DREAM study

## Abstract

**Background:**

Evidence suggests maternal birth experience impacts infant health. Alterations of the infant’s hypothalamus-pituitary-adrenal (HPA) axis are discussed as one possible underlying mechanism. This study aimed to investigate both objective and subjective birth experience as potential predictors of offspring’s hair glucocorticoid concentrations (GCs) and infant development, respectively. Further, we examined the role of hair GCs for prospective infant development in different domains.

**Methods:**

*n* = 263 mothers participating in the prospective cohort study DREAM_HAIR_ completed questionnaires about their objective and subjective birth experience approximately eight weeks after birth. Additionally, hair samples from *n* = 286 infants were taken around ten days (neonatal hair GCs) and eight weeks after birth (infant hair GCs) and long-term integrated hair cortisol and cortisone levels were measured in scalp-near 2-cm segments. Infant development (communication, gross motor, fine motor, problem-solving, personal-social) was assessed 14 months after birth using the Ages and Stages Questionnaire − 3 (ASQ-3).

**Results:**

No significant associations were found between objective or subjective birth experience and most domains of infant development, except that a more negative objective birth experience predicted poorer fine motor skills. Additionally, a more negative objective birth experience was linked to lower infant hair cortisone levels and a higher cortisol/cortisone ratio, while a more negative subjective experience was associated with higher neonatal hair cortisol. Lower neonatal hair cortisone showed a link to poorer personal-social development. However, after correction for multiple testing, only the associations between a more negative objective birth experience and lower hair cortisone and a higher cortisol/cortisone ratio at eight weeks remained significant.

**Conclusions:**

Objective aspects of the birth experience may have a more enduring impact on infant hair GCs than maternal subjective perceptions, particularly with higher birth complications being linked to lower infant cortisone and a higher infant cortisol/cortisone ratio. Given that this ratio may indicate reduced enzymatic activity in converting cortisol to its inactive form, results suggest that birth complications could affect the infant’s glucocorticoid metabolism. No robust associations were found between birth experiences or hair GCs and infant development. Further research in more diverse, at-risk populations is needed to clarify these complex relationships.

**Supplementary Information:**

The online version contains supplementary material available at 10.1186/s12887-025-05528-5.

## Introduction

Giving birth is a significant event in life, involving both physiological and psychological processes [1, 2] that can influence the future health of the infant [3, 4]. Recognizing the complexity of the birth experience is crucial to adopt a broader understanding of the concept, encompassing objective factors like unexpected medical interventions or prolonged labor, as well as the subjective perceptions of the birthing individual involved [5]. Studies indicate that a considerable number of women, with a prevalence rate of 7–34%, report a negative subjective birth experience [6], which has been linked to various maternal postpartum mental health issues such as anxiety, depression, and post-traumatic stress symptoms [7–10]. Furthermore, research has demonstrated that negative objective and subjective birth experiences can influence the quality of the mother-infant bond [6], which in turn may impact children’s development [11, 12]. However, there is a paucity of studies investigating the associations between objective and subjective birth experiences and infant development. While some research suggests that complications during birth, such as an unplanned cesarean section, can have adverse effects on infant development [13, 14 but see 15], research examining the association between birth experience and infant health outcomes is rare and often limited to single obstetric complications [16–18]. With the exception of Latva et al. [19], who found an association of negative maternal recollection of birth with more behavioral and emotional symptoms in six-year-old preterm born children, there exists a lack of prospective studies investigating how both maternal objective and subjective experiences during birth might affect infant development.

The prenatal and early postnatal phases are characterized by accelerated maturation of the endocrine, neural, and immune systems, pivotal in influencing and supporting infant growth and development [20]. In light of this, policymakers are increasingly emphasizing the significance of the “first 1000 days” and the age range of “0–3” years as crucial opportunities to impact child outcomes [21]. Therefore, early identification of risk factors during birth that may affect developmental progress is essential for fostering early childhood development. One underlying mechanism linking birth experience and infant development may be an alteration of the infant’s hypothalamic-pituitary-adrenal (HPA) axis. The activity of this primary stress regulatory system induces the secretion of glucocorticoid hormones (e.g., cortisol and cortisone), which in turn, affect various bodily functions including immune processes and adaptation to acute stress [22]. Previous evidence suggests an effect of maternal objective birth experiences on the development of her infant’s cortisol secretion. Specifically, results of studies using umbilical cord blood or neonate saliva to determine cortisol concentrations, indicate that complications during birth seem to be potential stressors for infants resulting in an altered endocrine stress response: For example, previous studies investigating the effect of mode of birth on infant cortisol levels have consistently shown that, compared with vaginally born infants, infants born by caesarean section (elective or emergency) have lower serum and cord cortisol levels taken immediately after birth [23–25], as well as lower salivary cortisol levels measured 6 months after birth [26], compared with vaginally born infants. Other studies have found that the highest cord blood cortisol levels are found in infants born by instrumental vaginal birth [24, 27]. These findings are in line with the hypothesis that non-instrumental vaginal birth represents a type of healthy stress (eustress) that plays a central role in the adaptation to the extrauterine environment due to physiological labour, which leads to the activation of, among others, the endocrine system [1, 2]. In contrast to the distress present during instrumental vaginal birth or cesarean section, which are associated with an altered stress response that may fall outside the normal range and which have been suggested to affect biobehavioral outcomes [28]. Additionally, the duration of the second stage of labor has been shown to be positively associated with umbilical cord cortisol levels [27]. The stress of being born has been suggested to modify the functioning of the offspring’s HPA axis, which can affect physical and mental health in the long-term [29, 30]. Koss and Gunnar [31] concluded in their research review that the early postnatal period is a critical time period for shaping the child’s HPA axis, although the direction in which infants’ cortisol secretion is modified by different early life stressors remains unclear.

Moreover, researchers have investigated potential associations between dysregulations of the HPA axis and infant development. However, findings are inconsistent: While some studies have found no significant links between (cord) blood or infants’ salivary cortisol and children’s motor and cognitive development [32, 33], others have reported significant associations. For instance, one study found that higher levels of evening infant salivary cortisol were linked to better cognitive and psychomotor development at 14 months, but this association was observed only in boys [34]. Another study reported that elevated evening infant salivary cortisol levels at 14 months were associated with lower socioemotional development in boys and with lower cognitive, gross motor development as well as executive function in girls at 4 years of age [35].

Inconsistent findings may be partly due to the fact that traditional cortisol methods in saliva, blood, or urine assess rather the short-term hormone secretion, which is heavily influenced by factors like ultradian and circadian rhythms or acute stress and are thought to offer limited insight into overall long-term systemic cortisol exposure [36–38]. In contrast, the analysis of hair glucocorticoid concentrations (GCs) has been proposed as a valid and reliable method for the assessment of cumulative glucocorticoid levels integrated over periods of several months [37], which should not be affected by situational variability (e.g., due to acute stress, diurnal rhythmicity; [37]). Until now, only a limited number of studies has examined the potential association between hair cortisol and infant development. One study demonstrated that elevated hair cortisol measured ten days after birth was predictive of impaired motor development at six months [39]. Another study found higher hair cortisol to be cross-sectionally associated with socio-emotional difficulties at one year [40].

Nevertheless, recent studies have challenged the theory that hair GCs accurately reflect a historical record of cumulative glucocorticoid levels over an extended time period. Instead, they propose an updated model of hair GCs as a marker sensitive to recent and ongoing physiological stress [41]. For example, in a study involving rats without adrenal glucocorticoid production, Colding-Jørgensen [42] demonstrated that corticosterone levels in hair rose within three hours of receiving corticosterone injections. The peak concentrations were observed on the seventh day of treatment, and a swift decline in concentrations post-treatment by day 26 implies that GCs detected in hair are not fixed but rather undergo diffusion in and out of the hair. In line with these findings, preliminary studies investigating the perinatal determinants of neonatal hair GCs measured in the first days after birth found that they were associated with prenatal exposures (e.g., maternal depressive symptoms, maternal GCs; [43, 44]), but also with birth-related characteristics (e.g., gestational age, presence of labor, birth weight) and postnatal exposures (e.g., neonatal sepsis; [44, 45]). Taken together, more research, also in humans, is needed to finalize an updated model of exactly what hair GC levels reflect. Based on the presented findings, we expected that while neonatal hair GCs may reflect long-term glucocorticoid exposure reaching into the intrauterine period, they are also significantly impacted by acute birth-related stressors, thus reflecting stress both within the intrauterine and extrauterine period.

The availability of glucocorticoids at the cellular level is regulated by tissue-specific metabolic enzymes, particularly 11β-hydroxysteroid dehydrogenases (11β-HSDs). Among these, 11β-HSD2 plays a crucial role as a potent dehydrogenase, efficiently converting cortisol into its inactive form, cortisone [46]. This conversion highlights the importance of both hair cortisol and hair cortisone as reliable biomarkers of systemic glucocorticoid levels [47, 48]. Additionally, the hair cortisol/cortisone ratio serves as an indirect measure of 11β-HSD2 enzymatic activity, providing insight into the balance between active and inactive glucocorticoids [49]. These biomarkers reflect long-term hypothalamic-pituitary-adrenal (HPA) axis activity and may be associated with birth-related variables and infant outcomes, as birth-related stress can influence the infant’s HPA axis function [3], potentially affecting long-term stress regulation and developmental outcomes [50, 51]. Therefore, evaluating hair cortisol, hair cortisone, and the hair cortisol/cortisone ratio may enhance our understanding of the associations between maternal subjective and objective birth experiences and offspring glucocorticoid secretion and metabolism, ultimately impacting infant development.

Therefore, the present prospective study aimed to expand the existing knowledge on associations of maternal objective and subjective birth experience with infant long-term HPA axis function and infant development. Given that maternal obstetric complications can adversely affect infant wellbeing and development [14], we hypothesized that negative objective birth experience was associated with adverse infant development measured in five domains (communication, gross motor, fine motor, problem-solving, personal-social) 14 months after birth. Initial studies suggest a predictive role of subjective birth experience for infant health [19]. Therefore, we expected an increase in explained variance by including subjective birth experience **(H1)**. Secondly, previous studies have demonstrated that stressful birth experiences can affect the cortisol secretion of the infant’s HPA axis [3]. Therefore, we hypothesized that the objective birth experience would be associated with offspring hair GCs, measured approximately ten days after birth representing long-term integrated biomarkers partly also affected by acute birth-related stress **(H2)** and eight weeks after the anticipated birth date as biomarkers of retrospective GCs (**H4**; [42, 52]). Given the complexity of the birth experience, we expected an increase in explained variance by including subjective birth experience. Due to heterogeneous prior results, no direction in which glucocorticoid secretion was modified by such early life stress was postulated [30, 53]. Third, the regulation of the infant’s HPA axis seems to be related to various later health problems [50, 51]. Accordingly, we hypothesized that hair GCs were associated with infant development measured in five domains 14 months after birth **(H3**,** H5)**. Again, due to the inconsistencies reported in previous studies, no direction was postulated. Finally, if birth experience was associated with hair cortisol, hair cortisone, or the hair cortisol/cortisone ratio, and the respective hair CG was shown to significantly prospectively predict infant development, then we conducted exploratory analyses to investigate whether the respective hair GCs mediated the association between birth experience and infant development.

The inconsistent previous evidence regarding the role of birth-related factors, GCs, and infant development may also be due to the fact that earlier studies have not systematically controlled for the influence of hair-related factors (e.g., natural hair color, storage time) on GCs. Furthermore, maternal prenatal depression has been shown to be associated with infant development [54, 55] Therefore in the present study, we controlled for both potential hair GCs confounders and maternal prenatal depressive symptoms to more clearly elucidate the relationship between birth experience, hair GCs, and infant development.

## Methods

### Study design and participants

The current study is part of the prospective, multi-method “Dresden Study on Parenting, Work, and Mental Health” (**DREAM**) investigating the effects of work, role distribution, perinatal factors, and stress factors on long-term family mental health and intra-family relationships. The study currently includes measurements at seven time points from pregnancy to early childhood (T1 DREAM – T7 DREAM). The endocrine sub-study DREAM_HAIR_ aims to investigate links between perinatal psychological stress, long-term steroid and endocannabinoid levels, and families’ mental and somatic health from pregnancy to 4.5 years after birth. General inclusion criteria for the main DREAM study were a current pregnancy, being resident in or around Dresden (Germany), and having sufficient German language skills. For DREAM_HAIR_, general exclusion criteria for offspring comprised having a severe disease which may potentially affect HPA axis activity (e.g., cerebral hemorrhage). For further details about the DREAM and DREAM_HAIR_ study, see Kress et al. [56].

**Fig. 1:**
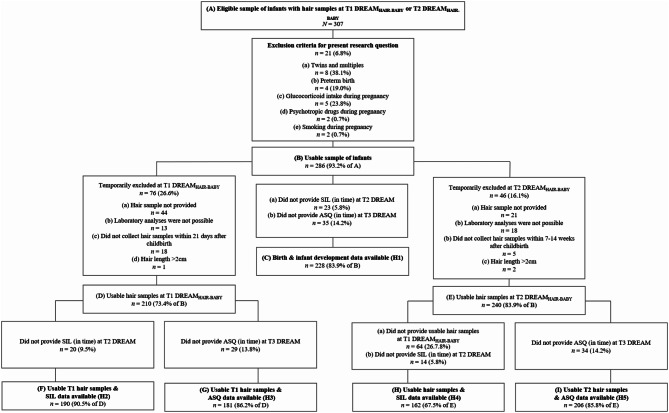
Flowchart of retention rate and exclusion criteria *Note*: Data used in this study were collected until January 31st, 2023 (data collection still ongoing, ninth version of the quality-assured dataset). SIL = Salmon’s Item List. ASQ-3 = Ages and Stages Questionnaire − 3

For the present investigation, questionnaire data were derived from (expectant) mothers at T1 DREAM (during pregnancy), T2 DREAM (eight weeks after the anticipated birth date), and T3 DREAM (14 months after the actual birth date). Additionally, infant hair samples from T1 DREAM_HAIR-BABY_ (assessed approximately ten days after birth) and T2 DREAM_HAIR-BABY_ (assessed approximately eight weeks after the anticipated birth date) were used to answer the research questions. The T2 data collection was scheduled eight weeks after the anticipated birth date provided in the T1 DREAM questionnaire. As the participants were not contacted between the first and second measurement time points, the actual birth date was not known until T2 DREAM, and subsequent questionnaires were adjusted accordingly. Specific exclusion criteria for the current study included multiple or preterm births, maternal use of glucocorticoids or psychotropic drugs during pregnancy, and regular smoking or alcohol consumption during pregnancy. We excluded hair samples at either T1 DREAM_HAIR-BABY_ or T2 DREAM_HAIR-BABY_ if laboratory analyses were not possible or if hair samples were not provided within a defined time frame (i.e., within 21 days after birth for T1 DREAM_HAIR-BABY_ or within 7—14 weeks after birth for T2 DREAM_HAIR-BABY_). For some research questions, infants had to be temporarily excluded if their mothers did not provide information on birth experience or infant development data (for further details, see Fig. 1).

We conducted attrition analyses regarding the main study variables and sociodemographic characteristics using *t*-tests and Fischer’s exact/chi-square test to assess whether completers (i.e., mother-infant dyads who completed T1 DREAM_HAIR-BABY_, T2 DREAM_HAIR-BABY_, T2 DREAM, and T3 DREAM; *n* = 161) differed from non-completers (i.e., participants who did not complete T3 DREAM; *n* = 15). Non-completers differed regarding country of birth, such that they were more likely than completers to report a country of birth other than Germany (*X*^*2*^ (1, *N* = 175) = 7.77, *p* =.005). Further, non-completers reported having a more positive objective birth experience than completers (*t*(174) = -3.13, *p* =.001). For all other study variables, there were no significant differences between completers and non-completers.

### Measurements

*Objective birth experience* was assessed at T2 DREAM using information from maternity records and self-administered questions [57]. Based on Garthus-Niegel et al. [9] and Jaramillo et al. [58], an index was calculated by summing the number of maternal complications and infant birth complications. The following complications were included: (1) No progress in the 2nd stage of labour, (2) active phase of labour lasted more than 12 h, (3) extensive blood loss, (4) vaginal, (5) labial, (6) perineal tear (3rd or 4th degree), (7) premature placental abruption, (8) difficulties with the placental abruption, (9) breech or (10) transverse presentation, 11) pathological heart sounds, 12) nuchal cord, 13) umbilical cord prolapse, 14) green amniotic fluid, 15) low Apgar score at 5 min (< 7). Furthermore, in light of previous findings investigating offspring cortisol levels, 16) vaginal or instrumental vaginal birth (as opposed to caesarean section) was incorporated into the index as a factor that is perceived to be more stressful for the infant [44]. Scores could range from 0 to 16, with higher scores indicating a more negative objective birth experience.

*Subjective birth experience* was assessed at T2 DREAM using the German version of Salmon’s Item List by Stadlmayr et al. (SIL; [59]). The SIL is a validated 20-item questionnaire that comprises the factors fulfilment, physical discomfort, emotional distress, and negative emotional experience. Participants were asked to rate how they felt during birth on a numeric scale (1 to 7) that captures antagonistic dimensions (e.g., disappointed – not disappointed). A total score ranging between 0 and 120 was generated, with lower scores indicating a more negative subjective birth experience. In the current sample, internal consistency was excellent (Cronbach’s α = 0.92).

*Infant development* was measured at T3 based on maternal report with the Ages and Stages Questionnaire – 3 (ASQ-3; 36). The ASQ is an age-graded, developmental screening instrument consisting of 30 items with six items in the five domains communication (ASQ_comm_), gross motor (ASQ_gross_), fine motor (ASQ_fine_), problem-solving (ASQ_problem_), and personal-social (ASQ_personal_). For the purpose of the DREAM study, the English version was translated into German and back-translated by a native speaker and adapted where necessary [60]. Parents are asked to mark *yes*, *sometimes*, or *not yet* according to their observation of the child’s behavior. Every yes answer is scored with 10 points, sometimes with 5 points, and not yet with 0 points, resulting in sum scores ranging from 0 to 60 for each domain. Squires et al. defined cut-off points for each domain (for 14 months version: ASQ_comm_: 17.4, ASQ_gross_: 25.8, ASQ_fine_: 23.06, ASQ_problem_: 22.56, ASQ_personal_: 23.18; [60]). Scores below the cut-offs suggest age-based development and more intensive follow-up assessments are recommended. In the current sample, internal consistency of the ASQ scales varied greatly between scales: Cronbach’s α_comm_ = 0.54, α_gross_ = 0.86, α_fine_ = 0.51, α_problem_ = 0.54, α_personal_ = 0.46.

*Infant hair GCs* (i.e., hair cortisol, hair cortisone) were assessed in scalp-near hair strands at T1 DREAM_HAIR_ (approximately ten days after birth) and T2 DREAM_HAIR_ (approximately eight weeks after the anticipated birth date). Based on the assumption that hair grows at a rate of approximately 1 cm per month [61], it can be inferred that segments of hair measuring ≤2 cm in length represent the integrated and cumulative cortisol levels over a maximum of the last two months. Hair samples were stored dry, dark, and at room temperature in aluminum foil before being sent in five batches to the biochemical laboratory at the Institute of Biological Psychology at the Faculty of Psychology of the Technische Universität Dresden (Prof. Clemens Kirschbaum). The analysis of hair GCs was conducted in accordance with a validated liquid chromatography-tandem mass spectrometry (LC-MS/MS) protocol [62].

*Potential confounding variables* that might influence hair GCs were considered: sex, gestational age, birth weight, and parity as assessed at T1 DREAM [63]; hair-related characteristics (e.g., frequency of washing, timepoint of hair sampling) and antibiotic intake [36] as assessed by an in-house hair protocol [64] in DREAM_HAIR_ at the time of hair sampling, as well as organizational factors (e.g., storage time of hair samples, batch effects). COVID-19 pandemic exposure was considered as a potential confounder to account for possible effects on hair GCs [65] and infant development [66, 67]. Participants were grouped into two categories with respect to the date of hair sampling and the date of assessment of infant development at T3 DREAM, respectively.

Further, for hair GCs at T1 DREAM_HAIR_, maternal hair GCs measured during pregnancy were regarded as potential confounding variables, as previous research suggests that neonatal hair cortisol may be associated with maternal hair cortisol during pregnancy [52] Hair samples from pregnant participants (T1 DREAM_HAIR_) were collected 4 ± 2 weeks before the anticipated birth date. The hair strands were taken from the posterior vertex of the head, in close proximity to the scalp. The same storage and analysis protocol as for infants’ hair samples was followed for the maternal hair samples (i.e., hair strands were stored in a dry and dark place at room temperature in aluminum foil, then sent to the Institute of Biological Psychology at the Technische Universität Dresden for analysis using a validated liquid chromatography-tandem mass spectrometry (LC-MS/MS) protocol [62].

*Prenatal depressive symptoms* were evaluated using the Edinburgh Postnatal Depression Scale (EPDS; [68–70]). This questionnaire consists of ten items, where respondents rate their symptoms during the past week on a scale from 0 to 3, yielding a total score ranging from 0 to 30. Higher scores indicate more severe symptoms. Previous research has highlighted the significance of depression during pregnancy for infant development [54, 55], so depressive symptoms assessed at T1 DREAM during pregnancy were accounted for as a potential influencing factor in analyses concerning infant development. The EPDS scores showed strong internal consistency (α = 0.84).

### Statistical analyses

All analyses were conducted using IBM SPSS Statistics 29. If items from psychometric scales were missing, they were replaced by the participant’s own mean score, calculated from their completed items, provided that at least 80% of the items were completed. For hair GCs data, non-detectable values were replaced by the lowest detectable value divided by two and if hair cortisol and hair cortisone lacked normality, data were log transformed to minimize biased results [71, 72]. Then, the hair cortisol/cortisone ratio was calculated by logarithmizing the quotient of hair cortisol and hair cortisone. For all hair GCs, outliers (i.e. values outside 3 *SD* ± mean) were replaced by the lowest or highest concentration that could be reliably detected in the sample (i.e. values within mean ± 3 *SD*; [73]).

We conducted descriptive analyses for sociodemographic characteristics and primary variables (i.e., objective and subjective birth experience, hair GCs, and infant development). Then, Spearman correlations were computed to gain information about the associations between all primary variables. Next, analyses were conducted to examine which confounders should be included in the main analyses: (1) Not all variables were normally distributed. Therefore, Spearman correlations were performed for T1 hair GCs and T2 hair GCs (i.e. hair cortisol, hair cortisone, hair cortisol/cortisone ratio) and potential confounders mentioned above. (2) To investigate whether batch effects and COVID-19 pandemic exposure were associated with T1 hair GCs and T2 hair GCs, we conducted hierarchical regression models with batch effects as dummy variables in Model 1 and COVID-19 pandemic exposure in Model 2. (3) The correlation between COVID-19 pandemic exposure and infant development, as well as (4) prenatal depressive symptoms and infant development was examined using Spearman rank correlation analysis. Variables that were significantly associated with hair cortisol, hair cortisone, hair cortisol/cortisone ratio, or infant development were implemented as confounders in main analyses with the respective variable as outcome (for full results of analyses including confounders, please refer to Additional files 1 and 2).

To investigate our research questions, (hierarchical) multiple regression analyses with 95% bias-corrected and accelerated (BCa) bootstrap confidence intervals with *N* = 2,000 bootstrap resampling procedures were conducted. For variable entry, the inclusion method was chosen. To test the first hypothesis, the effect of objective (Model 1) and then subjective birth experience (Model 2) on infant development was investigated respectively for each ASQ domain (ASQ_comm,_ ASQ_gross,_ ASQ_fine,_ ASQ_problem,_ ASQ_personal_). Then, after controlling for relevant confounders, the predictive value of objective (Model 1) and subjective birth experience (Model 2) for neonatal T1 DREAM_HAIR_ hair GCs (i.e., hair cortisol, hair cortisone, hair cortisol/cortisone ratio) as dependent variable was tested. Third, the respective T1 DREAM_HAIR_ hair GCs were investigated as predictors for infant development in each of the five ASQ domains. The above-mentioned analyses were also conducted for T2 DREAM_HAIR_ hair GCs measured eight weeks after the anticipated birth date. For the domain ASQ_personal,_ maternal prenatal depressive symptoms were included in Model 1, as they were a significant confounder.

If the results of the regression analyses indicated a potential mediating role of hair GCs in a specific domain, this was examined in detail with help of the SPSS modelling tool PROCESS [74], which uses ordinary least squares regression to compute unstandardized path coefficients of the total, direct, and indirect effect in a mediation model. Because regression analyses are sensitive to outliers, we identified multivariate outliers for hierarchical regressions using Mahalanobis distance [75] and excluded *n* = 5 outliers from our analyses to ensure more precise estimates. Therefore, results in the manuscript will be presented and discussed without the inclusion of the outliers (see characteristics of multivariate outliers in Additional files 3; see different results for analyses including multivariate outliers in Additional files 4). To address multiple testing, we applied the Šidák correction, which adjusts the significance threshold to control the familywise error rate. Specifically, we corrected for *m* = 15 comparisons, based on the highest number of tests conducted for a hypothesis (i.e., H3 and H5 involving three glucocorticoids and five ASQ domains). Using the Šidák correction formula, 1-(1-α)^(1/m), we set the adjusted significance threshold at a *p*-value of 0.003. Therefore, any *p*-value below 0.003 was considered statistically significant.

## Results

### Sample characteristics and baseline associations

The characteristics of the sample are shown in Table 1. Most of the mothers in this study were primiparous and were characterized by a rather high level of education. In terms of their objective birth experience, only 4.9% (*n* = 13) of mothers reported no birth complications. Approximately one quarter (*n* = 68, 25.9%) reported having one complication, while 34.2% (*n* = 90) had two. Approximately one in five participants experienced three birth complications (*n* = 59, 22.4%), 10.3% (*n* = 27) experienced four birth complications, and 2.3% of mothers reported more than five birth complications (*n* = 6).

Birth was considered a positive event by 69.2% (*n* = 175) of participants. The prevalence of negative subjective birth experiences was 30.8% (*n* = 78). Regarding infant characteristics, three children (1.2%) had scores below the cut-off on the ASQ_comm_ score, 42 children (16.7%) on the ASQ_gross_ scale, 10 children (4.0%) on the ASQ_fine_ scale, six (2.4%) on the ASQ_problem_ scale, and nine (3.6%) on the ASQ_personal_ scale.

Intercorrelations for the primary study variables are shown in Table 2. Subjective and objective birth experience were significantly correlated (*r* = −.28, *p* <.001), indicating that more birth complications were associated with lower SIL scores (i.e. a more negative subjective birth experience). A more negative objective birth experience was also associated with poorer ASQ_fine_ (*r* = −.14, *p* =.032), as well as with higher T1 hair cortisol, T2 hair cortisol (*r*’s > 0.18, *p*’s = 0.006), and the T2 hair cortisol/cortisone ratio (*r =*.25, *p* =.002). However, most associations did not survive Šidák correction. On the other hand, a more negative subjective birth experience was not associated with any ASQ scale (*r*’s < 0.02, *p*’s < 0.987), but was related to higher T1 hair cortisol, T1 hair cortisone, and T2 hair cortisol (*r*’s >|-0.16|, *p*’s < 0.029), although not below the threshold defined for the Šidák correction. Regarding hair GCs and infant development, a significant association was found between higher scores on ASQ_fine_ and higher T1 hair cortisone levels (*r* =.17, *p* =.026), as well as a lower T2 hair cortisol/cortisone ratio (*r* = −.14, *p* =.048). However, neither finding survived correction with a significant *p*-value of < 0.003.

**Table 1:** Sample characteristics of mothers and infants (n ^a^ = 263)

Variables	*n* (%) or Mean ± SD (range)
**Mother**	
**Sociodemographic characteristics**	
Age in years (*M*, *SD*, range) ^b^	29.97 ± 3.77 (18–42)
In a romantic relationship (*n*, %) ^b^	253 (98.4)
Primiparous (*n*, %) ^b^	219 (83.3)
University degree (*n*, %) ^b^	172 (65.4)
**Psychophysiological variables**	
Index objective birth experience (*M*, *SD*, range) ^c^	2.15 ± 1.17 (0–7)
Subjective birth experience (SIL; *M*, *SD*, range) ^c^	77.97 ± 21.64 (20–120)
Prenatal depressive symptoms (EPDS; *M*, *SD*, range) ^b^	5.46 ± 4.05 (0–19)
***Infant***	
**Sociodemographic characteristics**	
Sex ^c^	
Female (*n*, %) Male (*n*, %)	143 (55.9) 113 (44.1)
Gestational age at birth (weeks; *M*, *SD*, range) ^c^	40.46 ± 1.05 (38–42)
Birth weight (g; *M*, *SD*, range) ^c^	3401.67 ± 468.28 (1140–4890)
**Infant development (ASQ)** ^d^	
Communication (*M*, *SD*, range) Gross motor (*M*, *SD*, range) Fine motor (*M*, *SD*, range) Problem-solving (*M*, *SD*, range) Personal-social (*M*, *SD*, range)	44.19 ± 10.86 (12–60) 45.75 ± 17.22 (0–60) 43.31 ± 11.32 (5–60) 46.24 ± 11.01 (0–60) 46.09 ± 11.14 (15–60)
**Raw hair glucocorticoid concentrations**	
Hair cortisol (T1; pg/mg; *M*, *SD*, range) ^e^ Hair cortisol (T2; pg/mg; *M*, *SD*, range) ^f^ Hair cortisone (T1; pg/mg; *M*, *SD*, range) ^e^ Hair cortisone (T2; pg/mg; *M*, *SD*, range) ^f^ Hair cortisol/cortisone ratio (T1; pg/mg; *M*, *SD*, range) ^e^ Hair cortisol/cortisone ratio (T2; pg/mg; *M*, *SD*, range) ^f^	387.84 ± 215.12 (3.26–1042.88) 146.04 ± 108.01 (23.99–618.63) 176.79 ± 101.74 (39.49–574.11) 178.86 ± 143.21 (20.82–1146.27) 2.54 ± 1.49 (0.08–7.16) 1.32 ± 1.32 (0.12–8.88)

*Note*. EPDS = Edinburgh Postnatal Depression Scale; SIL = Salmon’s Item List; ASQ-3 = Ages and Stages Questionnaire – 3; Hair cortisol = hair cortisol concentrations; Hair cortisone = hair cortisone concentrations

^a^ Total n varies slightly due to missing values. ^b^ T1 DREAM (*M* = 26.78 pregnancy week, *SD* = 5.63). ^c^ T2 DREAM (*M* = 8.29 weeks after birth date, *SD* = 1.47). ^d^ T3 DREAM (*M* = 13.67 months after birth, *SD* = 0.47). ^e^ T1 DREAM_HAIR_ (*M* = 10.30 days after birth, *SD* = 4.08). ^f^ T2 DREAM_HAIR_ (*M* = 8.38 weeks after birth date, *SD* = 1.20)

**Table 2:** Spearman rank correlations between primary study variables (*n* = 162—228) ^a^

Variable	1	2	3	4	5	6	7	8	9	10	11	12	13
1. Index objective birth experience ^b^	-	− 0.28^**^	− 0.06	0.05	− 0.14^*^	0.01	− 0.06	0.20^*^	0.09	0.10	0.19^*^	− 0.15	0.25^**^
2. Subjective birth experience (SIL) ^b^		-	0.00	0.00	0.02	0.03	0.01	− 0.22^*^	− 0.16^*^	− 0.11	− 0.21^*^	− 0.03	− 0.11
3. ASQ_comm_^e^			-	0.13	0.33^**^	0.30^**^	0.39^**^	0.00	0.07	− 0.01	− 0.04	− 0.02	− 0.04
4. ASQ_gross_^e^				-	0.18^*^	0.32^**^	0.17^*^	− 0.01	0.10	0.00	− 0.06	0.04	− 0.07
5. ASQ_fine_^e^					-	0.48^**^	0.38^**^	0.03	0.17^*^	− 0.06	− 0.08	0.07	− 0.14^*^
6. ASQ_problem_^e^						-	0.31^**^	0.14	0.13	0.00	− 0.07	0.05	− 0.11
7. ASQ_personal_^e^							-	0.01	0.12	− 0.08	− 0.09	− 0.04	− 0.05
8. Neonatal HairF ^c^								-	0.34^**^	0.62^**^	0.46^**^	0.11	0.20^*^
9. Neonatal HairE ^c^									-	− 0.44^**^	0.00	0.44^**^	− 0.34^**^
10. Neonatal HairF/HairE ^c^										-	0.39^**^	− 0.24^**^	0.42^**^
11. Infant HairF ^d^											-	0.03	0.60^**^
12. Infant HairE ^d^												-	− 0.75^**^
13. Infant HairF/HairE ^d^													-

*Note*. SIL = Salmon’s Item List; HairF = Hair cortisol concentrations; HairE = Hair cortisone concentrations; ASQ-3 = Ages and Stages Questionnaire – 3. ASQ_comm_ = ASQ-3 communication domain. ASQ_gross_ = ASQ-3 gross motor domain. ASQ_fine_ = ASQ-3 fine motor domain. ASQ_problem_ = ASQ-3 problem-solving domain. ASQ_personal_ = ASQ-3 personal-social domain

Bootstrap results are based on 2,000 bootstrap iterations. Two-tailed testing.

^a^ Total n varies slightly due to exclusion criteria for individual research questions and missing values. ^b^ T2 DREAM (M = 8.29 weeks after birth date, SD = 1.47). ^c^ T1 DREAM_HAIR−BABY_ (M = 10.30 days after birth, SD = 4.08). ^d^ T2 DREAM_HAIR−BABY_ (M = 8.38 weeks after birth date, SD = 1.20). ^e^ T3 DREAM (M = 13.67 months after birth, SD = 0.47)

^*^p <.05 but did not survive Dunn-Sidàk correction (*p* >.003). ^**^p <.001

### Regression analyses

#### Objective and subjective birth experience as predictors of infant development 14 months after birth

Hierarchical regression analyses examining the predictive value of objective and subjective birth experience on infant development revealed that the standardized coefficients of objective birth experience and subjective birth experience failed to reach statistical significance for ASQ_comm,_ ASQ_gross_, ASQ_problem_, and ASQ_personal_ as outcome variables (β <|0.08|, *p’s* > 0.248; adj. *R*^*2*^*s* < 0.00). Only analyses with ASQ_fine_ as outcome variable showed that a negative objective birth experience (i.e., higher number of birth complications) was related to poorer ASQ_fine_ 14 months after birth (β = − 0.13, *p* =.045; *F*(1,225) = 3.80, *p* =.053, adj. *R*^*2*^ = 0.01; Model 1), however this association did not remain significant after Šidák correction. The inclusion of subjective birth experience did not significantly increase the variance explained (*ΔR*^*2*^ = 0.00; Model 2) and the predictor itself failed significance (β = − 0.03, *p* =.666).

#### Objective and subjective birth experience as predictors of neonatal and infant hair GCs

Using hierarchical regressions, we investigated the predictive value of objective and subjective birth experience on neonatal T1 hair GCs assessed approximately ten days after birth after controlling for relevant confounders. Results are shown in Table 3. First, analyses predicting *T1 hair cortisol* showed that after controlling for parity, gestational age, timepoint of hair sampling, batch effects, and maternal hair cortisol during pregnancy (*F*(5,171) = 8.89, *p* <.001, adj. *R*^*2*^ = 0.18; Model 1), a more negative objective birth experience predicted higher neonatal cortisol levels (β = 0.15, *p* =.026; *ΔF*(1,170) = 4.57, *p* =.034, *ΔR*^*2=*^ 0.02; Model 2). The standardized coefficient of objective birth experience lost significance when subjective birth experience was included, but the latter variable predicted higher neonatal cortisol levels (β = − 0.16, *p* =.042; *ΔF*(1,169) = 5.10, *p* =.025, *ΔR*^*2=*^ 0.02; Model 3), suggesting that a more negative subjective birth experience was associated with higher neonatal cortisol levels. Nevertheless, these findings did not withstand the Šidák correction, with a significant *p*-value of 0.003. Second, analyses predicting *T1 hair cortisone* showed that after controlling for gestational age and timepoint of hair sampling (*F*(2,175) = 4.81, *p* =.009, adj. *R*^*2*^ = 0.04; Model 1), neither objective birth experience (β = 0.09 *p* =.169; *ΔR*^*2=*^ 0.01; Model 2) nor subjective birth experience significantly predicted neonatal hair cortisone (β = − 0.14, *p* =.069; *ΔR*^*2=*^ 0.02; Model 3). Third, analyses with the *T1 hair cortisol/cortisone ratio* as the outcome showed that after controlling for gestational age, timepoint of hair sampling, and batch effects (*F*(3,183) = 5.32, *p* =.002, adj. *R*^*2*^ = 0.06; Model 1), the standardized coefficients of objective and subjective birth experience failed to reach statistical significance (β <|0.06|, *p’s* > 0.308) and failed to explain additional variance (*ΔR*^*2=*^ 0.00).

**Table 3:** Multiple hierarchical analyses predicting neonatal T1 DREAM_HAIR−BABY_ hair GCs (*n* = 190)

		T1 HairF ^a^						T1 HairE ^b^					T1 HairF/HairE ratio ^c^			
**Predictor**		β	*p*	[95% BCa CI]		*adj. R* ^*2*^		β	*p*	[95% Bca CI]	*adj. R*^*2*^.		β	*p*	[95% Bca CI]	*adj. R* ^*2*^
*Model 1*						0.18					0.04					0.08
Parity ^d^		− 0.20	0.002**	[-170.02, -47.40]				–	–	–			–	–	–	
Gestational age ^e^		0.21	0.008*	[13.10, 73.40]				0.17	0.020*	[0.10,0.07]			0.09	0.227	[-0.09, 0.33]	
Timepoint of hair sampling ^f^		− 0.15	0.055	[-15.43, 0.41]				− 0.15	0.024*	[0.00, 0.00]			− 0.16	0.037*	[-0.12, -0.03]	
Batch effects ^f^		− 0.23	0.001**	[-149.24, -42.94]				–	–	–			− 0.18	0.015	[-0.95, -0.14]	
Respective maternal hair GCs ^g^		0.06	0.344	[-42.52, 115.48]				–	–	–			–	–	–	
*Model 2*						0.20					0.04		–	–	–	0.08
Parity ^d^		− 0.17	0.006*	[-150.04, -27.05]				–	–	–			–	–	–	
Gestational age ^e^		0.21	0.007*	[15.13, 73.30]				0.16	0.024*	[0.00,0.07]			0.09	0.235	[-0.10, 0.33]	
Timepoint of hair sampling ^f^		− 0.14	0.075	[-14.47, 0.76]				− 0.15	0.022*	[0.00, 0.00]			− 0.16	0.044*	[-0.11, -0.02]	
Batch effects ^f^		− 0.25	< 0.001**	[-157.81, -50.05]				–	–	–			− 0.19	0.012*	[-0.98, -0.15]	
Respective maternal hair GCs ^g^		0.05	0.387	[-41.95, 111.80]				–	–	–			–	–	–	
Index objective birth experience ^e^		0.15	0.026*	[2.94, 54.10]				0.09	0.169	[0.00,0.04]			0.06	0.308	[-0.09, 0.23]	
*Model 3*						0.22					0.05					0.09
Parity ^d^		− 0.15	0.009*	[-144.64, -20.30]				–	–	–			–	–	–	
Gestational age ^e^		0.21	0.005*	[15.10, 71.99]				0.16	0.026*	[0.00,0.07]			0.09	0.240	[-0.09, 0.32]	
Timepoint of hair sampling ^f^		− 0.14	0.068	[-14.47, 0.76]				− 0.16	0.012*	[0.00, 0.00]			− 0.17	0.041*	[-0.11, -0.02]	
Batch effects ^f^		− 0.24	< 0.001	[-157.89, -46.49]				–	–	–			− 0.18	0.012	[-0.97, -0.13]	
Respective maternal hair GCs ^g^		0.08	0.234	[-37.94, 136.68]				–	–	–			–	–	–	
Index objective birth experience ^e^		0.10	0.172	[-10.11, 46.61]				0.04	0.534	[-0.02,0.04]			0.04	0.557	[-0.14, 0.23]	
Subjective birth experience (SIL) ^e^		− 0.16	0.042*	[-3.15, -0.10]				− 0.14	0.069	[0.00,0.00]			− 0.06	0.441	[-0.02, 0.01]	

*Note. Β =* Standardized beta coefficient. BCa CI = 95% bias corrected and accelerated bootstrap confidence interval (2,000 iterations), adj. *R*^*2*^. = Adjusted coefficient of

determination. SIL = Salmon’s Item List; HairF = hair cortisol concentrations; HairE = hair cortisone concentrations

^a^ Model 1: *F*(5, 171) = 8.89, *p* <.001; Model 2: Δ*F*(1,170) = 4.57, *p* =.034, Δ*R*^2^ = 0.02; Model 3: Δ*F*(1,169) = 5.10, *p* =.025, Δ*R*^2^ = 0.02

^b^ Model 1: *F*(2, 175) = 4.81, *p* =.009; Model 2: Δ*F*(1,174) = 1.35, *p* =.246, Δ*R*^2^ = 0.01; Model 3: Δ*F*(1,173) = 3.06, *p* =.082, Δ*R*^2^ = 0.02

^c^ Model 1: *F*(3, 183) = 5.32, *p* =.002; Δ*F*(1,182) = 0.75, *p* =.288, Δ*R*^2^ = 0.00; Model 3: Δ*F*(1,181) = 0.74, *p* =.391, Δ*R*^2^ = 0.00

^d^ Assessed at T1 DREAM (*M* = 26.78 pregnancy week, *SD* = 5.63). ^e^ Assessed at T2 DREAM (*M* = 8.29 weeks after birth date, *SD* = 1.47). ^f^ Assessed at T1 DREAM_HAIR−BABY_ (*M* = 10.30 days after birth, *SD* = 4.08). ^g^ Assessed at T1 DREAM_HAIR_ (*M* = 21.44 days before birth, *SD* = 10.36)

* *p* <.05 but did not survive Dunn-Sidàk correction (*p* >.003)

** p <.001

**Fig. 2:**
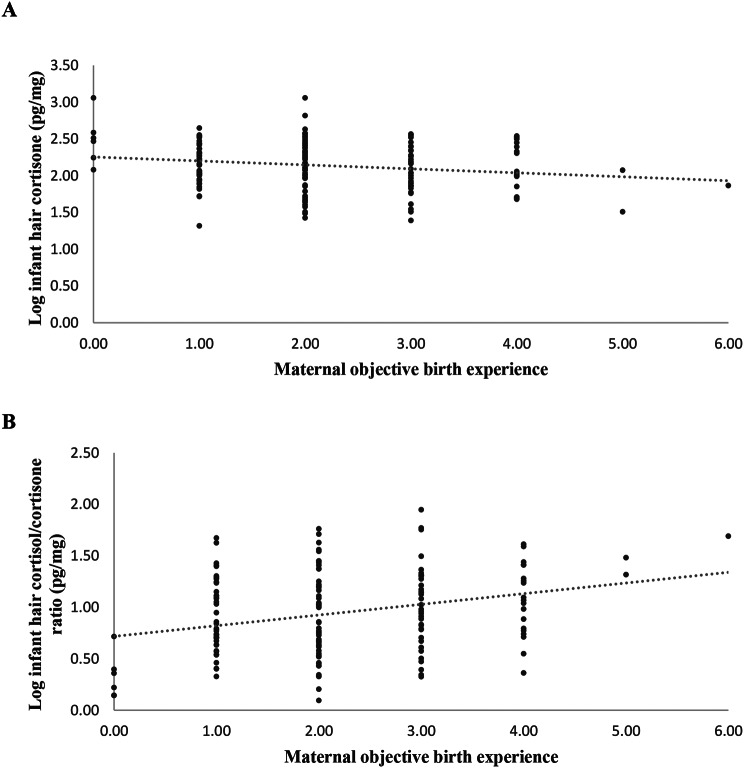
Scatterplots of statistically significant relationships between objective birth experience and infant hair GCs. *Note*: **A**) Scatterplot of the relationship between maternal objective birth experience and infant hair cortisone with a linear trendline (*n* = 160). **C**) Scatterplot of the relationship between maternal objective birth experience and infant hair cortisol/cortisone ratio with a linear trendline (*n* = 160)

We then conducted hierarchical regressions to investigate the predictive value of both objective and subjective birth experiences on infant T2 hair GCs, measured eight weeks after the anticipated birth date, while controlling for relevant confounders. The results are presented in Table 4. After controlling for T1 hair cortisol, parity, and gestational age in analyses predicting *T2 hair cortisol* (*F*(3,154) = 12.24, *p* <.001, adj. *R*^*2*^ = 0.18; Model 1), neither objective nor subjective birth experience were significant predictors (β <|0.14|, *p’s* > 0.082) or contributed to an increase in explained variance (*ΔR*^*2*^ < 0.02) in T2 hair cortisol. Moreover, after controlling for T1 hair cortisone, gestational age, natural hair color, and hair washing frequency (*F*(4,135) = 11.98, *p* <.001, adj. *R*^*2*^ = 0.24; Model 1), a more negative objective birth experience, was found to be a significant predictor of lower *T2 hair cortisone* (β = − 0.26, *p* <.001; *ΔF*(1,134) = 12.65, *p* <.001, *ΔR*^*2=*^ 0.06; Model 2), but subjective birth experience was not a significant predictor (β = − 0.07, *p* =.386; *ΔR*^*2=*^ 0.00; Model 3). Similarly, analyses predicting *T2 hair cortisol/cortisone* ratio showed that after controlling for T1 hair cortisol/cortisone ratio, natural hair color, and hair washing frequency, (*F*(3,136) = 15.51, *p* <.001, adj. *R*^*2*^ = 0.32; Model 1), a more negative objective birth experience predicted a higher T2 hair cortisol/cortisone ratio (β = 29, *p* <.001; *ΔF*(1,135) = 16.72, *p* <.001, *ΔR*^*2=*^ 0.08; Model 2). Subjective birth experience was not a significant predictor (β = − 0.03, *p* =.680; *ΔR*^*2=*^ 0.00; Model 3). Main results of statistically significant analyses with objective birth experience as predictor of infant hair GCs are graphically depicted in Fig. 2.

**Table 4:** Multiple hierarchical analyses predicting infant T2 DREAM_HAIR−BABY_ hair GCs (*n* = 162)

		T2 HairF ^a^					T2 HairE ^b^					T2 HairF/HairE ratio ^c^			
**Predictor**		β	*p*	[95% Bca CI]	*adj. R* ^*2*^		β	*p*	[95% Bca CI]	*adj. R* ^*2*^		β	*p*	[95% Bca CI]	*adj. R*^*2*^.
*Model 1*					0.18					0.24					0.24
T1 DREAM_HAIR−BABY_ hair GCs ^d^		0.39	< 0.001**	[0.00, 0.00]			0.40	< 0.001**	[0.39, 0.88]			0.39	< 0.001**	[0.07, 0.16]	
Parity ^e^		− 0.05	0.369	[-0.11, 0.04]			–	–	–			–	–	–	
Gestational age ^f^		0.09	0.203	[-0.01, 0.06]			0.07	0.355	[-0.03, 0.08]			–	–	–	
Natural hair color ^g^		–	–	–			− 0.14	0.058	[-0.20, -0.00]			0.15	0.038*	[0.10, 0.24]	
Hair washing frequency ^g^		–	–	–			− 0.20	0.005*	[-0.15, -0.03]			0.24	. <001**	[0.05, 0.19]	
*Model 2*					0.19					0.30					0.32
T1 DREAM_HAIR−BABY_ hair GCs ^d^		0.37	<0.001**	[0.00, 0.00]			0.43	< 0.001**	[0.42, 0.93]			0.36	< 001**	[0.07, 0.15]	
Parity ^e^		− 0.03	0.566	[-0.10, 0.06]			–	–	–			–	–	–	
Gestational age ^f^		0.09	0.179	[-0.01, 0.06]			0.08	0.260	[-0.02, 0.08]			–	–	–	
Natural hair color ^g^		–	–	–			− 0.14	0.042*	[-0.19, -0.01]			0.15	0.025*	[0.02, 0.23]	
Hair washing frequency ^g^		–	–	–			− 0.19	0.009*	[-0.14, -0.03]			0.23	0.002**	[0.05, 0.19]	
Index objective birth experience ^f^		0.12	0.108	[-0.01, 0.06]			− 0.26	< 0.001**	[-0.11, -0.03]			0.29	< 0.001**	[0.05, 0.15]	
*Model 3*					0.20					0.30					0.31
T1 DREAM_HAIR−BABY_ hair GCs ^d^		0.36	< 0.001**	[0.00, 0.00]			0.42	< 0.001**	[0.41, 0.92]			0.36	< 0.001**	[0.06, 0.15]	
Parity ^e^		− 0.02	0.821	[-0.09, 0.08]			–	–	–			–	–	–	
Gestational age ^f^		0.09	0.161	[-0.01, 0.06]			0.08	0.232	[-0.02, 0.08]			–	–	–	
Natural hair color ^g^		–	–	–			− 0.14	0.045*	[-0.19, -0.01]			0.15	0.023*	[0.02, 0.23]	
Hair washing frequency ^g^		–	–	–			− 0.20	0.006*	[-0.14, -0.03]			0.23	. 001**	[0.04, 0.18]	
Index objective birth experience ^f^		0.08	0.296	[-0.02, 0.05]			− 0.27	< 0.001**	[-0.12, -0.04]			0.28	< 0.001**	[0.04, 0.14]	
Subjective birth experience (SIL) ^f^		− 0.14	0.082	[0.00, 0.00]			− 0.07	0.386	[-0.00,0.00]			− 0.03	0.680	[00.0, 0.00]	

*Note. Β =* Standardized beta coefficient. Bca CI = 95% bias corrected and accelerated bootstrap confidence interval (2,000 iterations), adj. *R*^*2*^. = Adjusted coefficient of

determination. SIL = Salmon’s Item List; HairF = hair cortisol concentrations; HairE = hair cortisone concentrations

^a^ Model 1: *F*(3, 154) = 12.24, *p* <.001; Model 2: Δ*F*(1,153) = 2.60, *p* =.109, Δ*R*^2^ = 0.01; Model 3: Δ*F*(1,152) = 3.23, *p* =.074, Δ*R*^2^ = 0.02

^b^ Model 1: *F*(4, 135) = 11.98, *p* <.001; Model 2: Δ*F*(1,134) = 12.65, *p* <.001, Δ*R*^2^ = 0.06; Model 3: Δ*F*(1,133) = 0.73, *p* =.396, Δ*R*^2^ = 0.00

^c^ Model 1: *F*(3, 136) = 15.51, *p* <.001; Model 2: Δ*F*(1,135) = 16.72, *p* <.001, Δ*R*^2^ = 0.08; Model 3: Δ*F*(1,134) = 0.181, *p* =.672, Δ*R*^2^ = 0.00

^d^ Assessed at T1 DREAM_HAIR−BABY_ (*M* = 10.30 days after birth, *SD* = 4.08). ^e^ Assessed at T1 DREAM (*M* = 26.78 pregnancy week, *SD* = 5.63). ^f^ Assessed at T2 DREAM (*M* = 8.29 weeks after birth date, *SD* = 1.47). ^g^ T2 DREAM_HAIR_ (*M* = 8.38 weeks after birth date, *SD* = 1.20)

* *p* <.05 but did not survive Dunn-Sidàk correction (*p* >.003)

** p <.001

#### Neonatal and infant hair GCs as predictors of infant development 14 months after birth

We also investigated the predictive value of neonatal and infant hair GCs for infant development in five domains (ASQ_comm,_ ASQ_gross,_ ASQ_fine,_ ASQ_problem,_ ASQ_personal_) at 14 months. None of the T1 nor T2 hair GCs significantly predicted infant development measured with the five ASQ scales (ß <|-0.08|, *p’s* > 226; adj. *R*^*2*^*s* < 0.00), with the exception of T1 hair cortisone predicting ASQ_personal_. Specifically, after controlling for prenatal maternal depressive symptoms (*F*(1,175) = 1.63, *p* =.203; adj. *R*^*2*^ = 0.00; Model 1), lower neonatal hair cortisone predicted lower ASQ_personal_ (ß =. 13, *p* =.044; *ΔF*(1,174) = 3.48, *p* =.064; *ΔR*^*2=*^ 0.02; Model 2), however the finding did not survive Šidák correction.

## Discussion

The present study prospectively examined whether both objective and subjective birth experiences could predict neonatal and infant hair GCs (i.e., hair cortisol, hair cortisone, and hair cortisol/cortisone ratio) and infant development, while accounting for relevant confounders. Further, we investigated the role of hair GCs in predicting infant development across different domains. Initial analyses suggested that a more negative objective and subjective birth experience measured approximately eight weeks after the anticipated birth date did not significantly predict infant development at 14 months, with one exception: a more negative objective birth experience was linked to poorer fine motor development. Further, a more negative objective birth experience predicted lower infant hair cortisone, and a higher infant hair cortisol/cortisone ratio measured eight weeks after the anticipated birth date. A more negative subjective birth experience was found to significantly predict higher neonatal hair cortisol. When examining hair glucocorticoids as predictors, most associations were not significant, except that lower neonatal hair cortisone was linked to poorer personal-social development. However, given the multiple comparisons conducted, we applied a correction for multiple testing to reduce the risk of false positives. After this correction, most observed effects were no longer statistically significant (adjusted *p* >.003). The only associations in regression analyses that survived correction were between a more negative objective birth experience and both lower infant hair cortisone levels and a higher hair cortisol/cortisone ratio, measured eight weeks after the expected birth date. Thus, while the findings will be carefully discussed, they should be interpreted with caution.

### Birth experience and infant development

Contrary to our initial hypotheses, there were no statistically significant associations found between either the objective or subjective birth experiences and infant development at 14 months in the domains of communication, gross motor, problem-solving, nor personal-social development. Unlike prior studies that focused on individual birth complications’ predictive effects on infant health, we utilized a cumulative index combining various objective birth complications, aligning with recommendations from several researcher groups [4, 76]. We only found a small negative effect of objective birth experience on fine motor development at 14 months. Although results did not survive correction for multiple testing, they are consistent with previous evidence focusing on individual birth complications and infant development, showing that breech presentation, or a low Apgar score [17, 18] increase the risk for poor psychomotor development and neurodevelopmental delay [77]. Given that fine motor skills have been linked to other key aspects of learning and development, including executive functions, adequate functioning in life and school performance [78, 79], it may be beneficial to proactively support the development of fine motor skills in infants who experience mild birth complications, even when the effects on motor outcomes are subtle and not consistently detected. Further, (the lack of) significant results with the ASQ scales as outcome variable should be interpreted with caution due the low reliability observed in our study. The internal consistency examined by the authors of the ASQ-3, based on completion by 18,000 respondents, ranged from 0.60 to 0.87 for the 14-month ASQ interval [60], compared with 0.46 to 0.86 in our sample. It is possible that there was limited variability in infant development scores in our relatively healthy sample. Although in terms of the subjective birth experience, our findings parallel those of Latva et al. who similarly found no association between negative maternal recollections of birth and behavioral and emotional symptoms in six-year-old term children [19], the association between a more negative objective and subjective birth experience child development (especially fine motor development) need to be investigated in more diverse and affected samples before firm conclusions can be drawn.

### Birth experience and neonatal and infant long-term hair GCs

Given new studies challenging the notion that hair GCs are an accurate marker for cumulative glucocorticoid levels [41, 42] and preliminary studies showing that neonatal hair GCs collected within the first few days after birth may be influenced by short-term birth-related stress in addition to intrauterine cortisol regulation [44, 45], we hypothesized that birth experience would be predictive of neonatal hair GCs assessed approximately ten days after birth. Overall, most initial analyses indicated no association between a negative birth experience and hair GCs, with the exception of cortisol. Specifically, correlation analyses showed that a more negative objective birth experience was linked to higher neonatal hair cortisol, consistent with previous studies (e.g., [24, 27]). However, this association did not hold in multiple regression analyses. In the final hierarchical regression model, the predictive value of objective birth experience was no longer significant after accounting for other relevant predictors and subjective birth experience. This shift in significance underscores the complex interplay and shared variance between objective and subjective birth experiences. Specifically, a more negative subjective birth experience emerged as a significant predictor of higher neonatal hair cortisol levels. Although correlation analyses were also significant, the predictive value of subjective birth experience did not survive Šidák correction for multiple comparisons in the regression analysis (threshold *p* <.003). Given the significant correlations between objective and subjective birth experience and neonatal cortisol (although not robust after correction), these findings suggest the need for further investigation to better understand the relative contributions of objective and subjective birth experiences to neonatal stress markers. Further, we suggest that future studies consider these birth-related aspects when investigating the effects of prenatal stress on intrauterine glucocorticoid regulation.

We then tested the hypothesis that maternal birth experience would prospectively also predict infant hair GCs measured eight weeks after the anticipated birth date after controlling for relevant confounders, including neonatal hair GCs. Contrary to our hypothesis, we did not find significant associations between a more negative objective birth experience and infant hair cortisol as compared to previous research showing correlations between single birth complications (e.g., neonatal sepsis) and hair cortisol [44]. However, we did find a significant association between a more negative objective birth experience and *lower infant hair cortisone* as well as higher *infant hair cortisol/cortisone ratio*, indicating that a higher number of birth complications was linked to reduced cortisone levels and a higher cortisol/cortisone ratio. This pattern of findings of GCs and their ratio in neonatal hair has been also reported in other studies [44, 45]. Previous research has shown that the hair cortisol/cortisone ratio can be used as an indirect marker of enzymatic activity of 11β-HSD2 [49]. This enzyme oxidizes cortisol into inactive cortisone and is therefore relevant for rendering the mineralocorticoid receptor-hormone complex inactive. Currently, there is limited research on the role of the cortisol/cortisone ratio in infant health, with existing studies primarily focusing on physiological outcomes. For instance, De Jong et al. observed a significantly higher cortisol/cortisone ratio in very low birthweight infants during the first two years compared to full-term children [80]. Wirix et al. reported that an elevated ratio was linked to an increased risk of hypertension in overweight children [81]. Modifications in the rate of conversion of cortisol to cortisone can result in an altered equilibrium, leading to an increased availability of cortisol and a decrease in the absolute concentrations of cortisone [82]. This could potentially explain the correlation between a higher number of birth complications and lower hair cortisone levels. Nevertheless, further investigation is necessary to elucidate these findings.

### Neonatal and infant long-term integrated GCs and infant development

Analyses investigating the predictive role of neonatal and infant hair GCs on infant development at 14 months revealed no significant associations between neonatal or infant hair GCs and any developmental ASQ domains, with the exception of lower neonatal hair cortisone predicting lower personal-social development. Findings should be interpreted cautiously, since they did not survive Šidák correction, however, they align with prior research highlighting the critical role of disruptions in glucocorticoid metabolism [83] that could later impact the developing brain, potentially leading to enduring effects on behaviour and cognitive function [84]. Lower levels of cortisone signify diminished concentrations of the inactive form of cortisol. Maintaining a healthy balance in cortisol and cortisone levels appears crucial for optimal development, as the effective availability of GCs in cell cytoplasm centres on the equilibrium between the active and inactive forms of these hormones [85].

### Strengths and limitations

This investigation is one of the first studies considering both objective as well as subjective birth experience and offspring hair GCs to predict infant development. Prospective-longitudinal data from the cohort study DREAM was used, which is a major strength of this investigation. Therefore, it was possible to measure predictor and outcome variables at different time points and examine longitudinal associations starting from birth until 14 months after birth. Additionally, the study stands out in the research field of birth experiences and infant outcomes by evaluating a robust glucocorticoid index through simultaneous examination of hair cortisol, hair cortisone, and the hair cortisol/cortisone ratio. Also, we considered several potential confounders that could have influenced hair GCs and infant development, including maternal hair GCs, and maternal depressive symptoms.

However, the study has some limitations. Firstly, the sample used was a community sample consisting of infants born to highly educated parents mostly born in Germany, most of whom were expecting their first child. Most of the infants in this study had relatively high scores on the developmental scales, well above the clinically relevant cut-offs, indicating a relatively healthy sample of infants. Further, it is a limitation that the German version of the ASQ-3 we used has not been validated yet. Also, unlike the original version of the ASQ-3, our instructions did not include a directive for parents to actively assess their child’s ability to perform the tasks outlined in the questionnaire items. This modification was implemented for the purpose of our study, to enable parents to fill out our questionnaires without the child necessarily being around. However, we acknowledge that this departure from the standard protocol may have led to a more subjective perception of the child’s competencies provided by parents and affected the psychometric characteristics of the scale and emphasize the need for cautious interpretation of findings derived from scales with low reliability (i.e., the association between objective birth experience and fine motor, as well as neonatal hair cortisone with personal-social development).The study’s design included a 14-month gap between birth and the assessment of children’s development, which raises the possibility that other risk factors may have influenced the child’s development during the first year of life. Adverse birth experiences can lead to impaired mother-infant bonding [10, 86, 87] or parental mental health problems [5, 10], both of which are known to affect infant development in the postnatal period [30]. It is of the utmost importance to identify any additional risk factors and to gain an understanding of their associations with both subjective and objective aspects of the birth experience, in order to clarify the links with infant development.

## Conclusion

In this prospective study, a more negative objective and subjective birth experience did not predict infant development across communication, gross motor, problem-solving, and personal-social skills, with the exception that a more negative objective birth experience was linked to poorer fine motor development. Additionally, we found that a more negative subjective birth experience was associated with higher neonatal hair cortisol levels measured around ten days after birth, while a more negative objective birth experience was linked to lower hair cortisone levels and a higher hair cortisol/cortisone ratio at eight weeks postpartum. Although most associations between hair glucocorticoids (GCs) and infant development were not robust, there was a link between lower neonatal hair cortisone and poorer personal-social development. However, after correction for multiple testing, only the associations between a more negative objective birth experience and both lower hair cortisone and a higher cortisol/cortisone ratio at eight weeks remained significant.

Taken together, these findings suggest that objective aspects of the birth experience may have a more enduring impact on infant stress markers, while the role of hair GCs in predicting infant development remains inconclusive. Further research is needed to clarify these complex relationships.

## Electronic supplementary material

Below is the link to the electronic supplementary material.

Supplementary Material 1

## Data Availability

The datasets used and/or analyzed during the current study are available from the corresponding author on reasonable request.

## Abbreviations

HPA-axis: Hypothalamus-pituitary-adrenal axis
GCs: Glucocorticoid concentrations
11β-HSD2: 11β-hydroxysteroid-dehydrogenase type 2
