# Supplementary material for: Infant development at 14 months in the context of maternal objective and subjective birth experience and infant hair glucocorticoids
Source: BMC Pediatr. 2025 Mar 14;25:191. doi: 10.1186/s12887-025-05528-5 (PMC11907962; doi:10.1186/s12887-025-05528-5)
Supplement: Supplementary file 1 — Supplementary Material 1 [file 12887_2025_5528_MOESM1_ESM.docx]

**Additional files for the manuscript “****Infant development at 14 months in the context of maternal objective and subjective birth experience and infant hair glucocorticoids”**

# **Additional file 1. Potential confounding variables in hair glucocorticoid concentrations**

| **Supplementary Table 1.1** | | | | | | |
| --- | --- | --- | --- | --- | --- | --- |
| *Results of Spearman correlations between T1 DREAM_HAIR_ concentrations and potentially confounding variables (n = 189)* | | | | | | |
| **Variables** | ***HairF***  ***β***  ***t***  ***.15***  ***2.33****  ***.22***  ***3.49******  ***-.20***  ***-3.14***** | | ***HairE*** | | ***HairF/HairE ratio*** | |
|  | Spearman | *p* | Spearman | *p* | Spearman | *P* |
| Sex | .08 | *.288* | .11 | *.153* | -.05 | *.518* |
| Gestational age | **.32** | *<.001* | **.20** | *.007* | .**19** | *.008* |
| Birth weight | .04 | *.554* | .05 | *.478* | .04 | *.548* |
| Parity | **-.25** | *<.001* | -.14 | *.057* | -.09 | *.213* |
| Hair color ^a^ | .11 | *.156* | .02 | *.844* | .07 | *.383* |
| Frequency of washing | -.15 | *.051* | -.11 | *.149* | -.01 | *.943* |
| Antibiotic intake | -.02 | *.761* | -.13 | *.079* | .04 | *.629* |
| Storage time | -.13 | *.068* | **-.19** | *.011* | .02 | *.806* |
| Timepoint of hair sampling | **-.26** | *<.001* | .005 | *.950* | **-.26** | *<.001* |
| Maternal HairF | **.15** | *.049* | - | *-* | **-** | *-* |
| Maternal HairE | - | *-* | .06 | *.407* | - | *-* |
| Maternal HairF/HairE ratio | - | *-* | **-** | *-* | -.01 | *.936* |
| *Note.*  HairF = hair cortisol concentrations; HairE = hair cortisone concentrations; Correlation coefficients for HairF, log-transformed HairE, and the HairF/HairE ratio are reported. Two tailed testing.  ^a^ Hair color was dichotomized into 1= Light (blonde, dark blonde, reddish blonde) and 2 = Dark (red, light brown, brown, dark brown, black).  * Significant correlation coefficients are marked in bold. | | | | | | |

| **Supplementary Table 1.2**  *Results of multiple hierarchical regressions predicting T1 DREAM_HAIR_ glucocorticoid concentrations (n = 190)* | | | | | | | | |
| --- | --- | --- | --- | --- | --- | --- | --- | --- |
|  | |  | **Hair F** | | **HairE** | | **HairF/HairE ratio** | |
| Model | Predictor | | ß | *p* | ß | *p* | ß | *p* |
| **1** | Batch 2 vs. Batch 1 | | **-.36** | <.001 | -.15. | .090 | **-.19** | .021 |
|  | Batch 3 vs. Batch 1 | | -.11 | .197 | -.04 | .602 | -.02 | .799 |
|  | Batch 4 vs. Batch 1 | | -.09 | .247 | -.14 | .070 | .09 | .216 |
|  | Batch 5 vs. Batch 1 | | -.09 | .185 | -.06 | .430 | -.04 | .559 |
| **2** | Batch 2 vs. Batch 1 | | **-.36** | <.001 | -.15 | .090 | **-.20** | .021 |
|  | Batch 3 vs. Batch 1 | | -.08 | .593 | -.15 | .352 | .03 | .838 |
|  | Batch 4 vs. Batch 1 | | -.07 | .538 | -.21 | .077 | .13 | .264 |
|  | Batch 5 vs. Batch 1 | | -.09 | .284 | -.09 | .289 | -.03 | .751 |
|  | COVID-19 pandemic exposure | | -.03 | .847 | .13 | .440 | -.07 | .685 |
| *Note.*  HairF = hair cortisol concentrations; HairE = hair cortisone concentrations; Regression coefficients for HairF, log-transformed HairE, and the HairF/HairE ratio are reported.  * Significant standardized coefficients are marked in bold. | | | | | | | | |

| **Supplementary Table 1.3** | | | | | | |
| --- | --- | --- | --- | --- | --- | --- |
| *Results of Spearman correlations between T2 DREAM_HAIR_ glucocorticoid concentrations and potentially confounding variables (n = 162)* | | | | | | |
| **Variables** | ***HairF***  ***β***  ***t***  ***.15***  ***2.33****  ***.22***  ***3.49******  ***-.20***  ***-3.14***** | | ***HairE*** | | ***HairF/HairE ratio*** | |
|  | Spearman | *p* | Spearman | *p* | Spearman | *P* |
| Sex | -.06 | *.466* | .14 | *.076* | -.14 | *.078* |
| Gestational age | **.19** | *.017* | **.19** | *.014* | -.04 | *.580* |
| Birth weight | .03 | *.718* | .03 | *.732* | .01 | *.924* |
| Parity | **-.19** | *.012* | -.01 | *.955* | -.11 | *.163* |
| Hair color | .03 | *.743* | **-.16** | *.049* | **.17** | *.044* |
| Frequency of washing | .03 | *.670* | **-.26** | *.005* | **.23** | *.004* |
| Antibiotic intake | .08 | *.338* | -.09 | *.241* | .11 | *.170* |
| Storage time | -.01 | *.954* | -.04 | *.615* | .05 | *.569* |
| Timepoint of hair sampling | -.13 | *.095* | -.05 | *.523* | -.03 | *.682* |
| T1 HairF | **.46** | *<.001* | - | *-* | **-** | *-* |
| T1 HairE | - | *-* | **.44** | *<.001* | - | *-* |
| T1 HairF/HairE ratio | - | *-* | - | *-* | **.42** | *<.001* |
| *Note.*  HairF = hair cortisol concentrations; HairE = hair cortisone concentrations; Correlation coefficients for log-transformed HairF, HairE, and the HairF/HairE ratio are reported. Two tailed testing.  * Significant correlation coefficients are marked in bold. | | | | | | |

| **Supplementary Table 1.4**  *Results of multiple hierarchical regressions predicting T2 DREAM_HAIR_ glucocorticoid concentrations (n = 161)* | | | | | | | | |
| --- | --- | --- | --- | --- | --- | --- | --- | --- |
|  | |  | **HairF** | | **HairE** | | **HairF/HairE ratio** | |
| Model | Predictor | | ß | *p* | ß | *p* | ß | *p* |
| **1** | Batch 2 vs. Batch 1 | | -.17 | .069 | -.15 | .121 | .00 | .966 |
|  | Batch 3 vs. Batch 1 | | .10 | .305 | -.06 | .541 | .11 | .237 |
|  | Batch 4 vs. Batch 1 | | .08 | .339 | -.12 | .157 | .16 | .074 |
| **2** | Batch 2 vs. Batch 1 | | -.17 | .069 | -.15 | .122 | .00 | .966 |
|  | Batch 3 vs. Batch 1 | | .21 | .475 | .04 | .884 | .11 | .719 |
|  | Batch 4 vs. Batch 1 | | .16 | .443 | -.05 | .798 | .15 | .471 |
|  | COVID-19 pandemic exposure | | -.13 | .681 | -.12 | .720 | .01 | .987 |
| *Note.*  HairF = hair cortisol concentrations; HairE = hair cortisone concentrations; Regression coefficients for HairF, log-transformed HairE, and the HairF/HairE ratio are reported.  * Significant standardized coefficients are marked in bold. | | | | | | | | |

# **Additional file 2. Potential confounding variables in infant development**

| \| **Supplementary Table 2** \| \| --- \| \| *Results of Spearman correlations between infant development and potentially confounding variables (n = 242)* \| | | | | | | | | | | |
| --- | --- | --- | --- | --- | --- | --- | --- | --- | --- | --- | --- | --- |
| Variables | ASQ_comm_ | | ASQ_gross_ | | ASQ_fine_ | | ASQ_problem_ | | ASQ_personal_ | |
|  | r_s_ | *p* | r_s_ | *p* | r_s_ | *p* | r_s_ | *p* | r_s_ | *p* |
| Prenatal depressive symptoms | .03 | *.613* | -.01 | *.913* | .09 | *.169* | .06 | *.331* | **.15** | *.022* |
| COVID-19 pandemic exposure | -.03 | *.671* | .06 | *.332* | -.04 | *.522* | -.058 | *.372* | -.04 | *.545* |
| *Note.*  ASQ_comm_ = Communication scale of the Ages and Stages Questionnaire– 3. ASQ_gross_ = Gross motor scale of the Ages and Stages Questionnaire– 3. ASQ_fine_ = Fine motor scale of the Ages and Stages Questionnaire– 3. ASQ_problem_ = Problem-solving scale of the Ages and Stages Questionnaire– 3. ASQ_personal_= Personal-social scale of the Ages and Stages Questionnaire– 3. Correlation coefficients are reported. Two tailed testing | | | | | | | | | | |

**Additional file 3. Outlier characteristics**

**Supplementary Table 3**

*Multivariate outlier identified by Mahalanobis Distance*

| Multivariate Outlier | OBE | SBE | ASQ_comm_ | ASQ_gross_ | ASQ_fine_ | ASQ_problem_ | ASQ_personal_ | T1 HairF | T1 HairE | T1 HairF/HairE | T2 HairF | T2 HairE | T2  HairF/HairE |
| --- | --- | --- | --- | --- | --- | --- | --- | --- | --- | --- | --- | --- | --- |
| 1 | 4.00 | 88.00 | 50.00 | 20.00 | 40.00 | 40.00 | 45.00 | 3.26 | 39.49 | .08 | 122.28 | 204.06 | .60 |
| 2 | 3.00 | 104.00 | 25.00 | 30.00 | 25.00 | 10.00 | 60.00 | 802.77 | 141.75 | 5.66 |  |  |  |
| 3 | 2.00 | 85.00 | 30.00 | 35.00 | 30.00 | 50.00 | 60.00 | 4.09 | 39.49 | .10 | 238.90 | 59.36 | 4.02 |
| 4 | 3.00 | 103.00 | 30.00 | .00 | 20.00 | .00 | 20.00 |  |  |  | 23.99 | 22.94 | 1.05 |
| 5 | 7.00 | 86.00 | 55.00 | 5.00 | 35.00 | 40.00 | 50.00 |  |  |  | 206.52 | 272.25 | .76 |
| *Note*. OBE = objective birth experience; SBE = subjective birth experience; HairF = hair cortisol concentrations; HairE = hair cortisone concentrations | | | | | | | | | | | | | |

# **Additional file 4. Results from hierarchical regression analyses including multivariate outliers (showing different results than hierarchical regression analyses excluding multivariate outliers)**

**Supplementary Table 4.1**

*Multiple hierarchical analyses predicting neonatal* ASQ_personal_ *(n =*179*)*

| **Predictor** |  | ß | *p* | [95% BCa CI] | adj. *R^2^* |
| --- | --- | --- | --- | --- | --- |
| **Model 1** |  |  |  |  | .01 |
| T1 EPDS ^a^ |  | .11 | .127 | [-0.08,0.67] |  |
| **Model 2** |  |  |  |  | .02 |
| T1 EPDS ^a^ |  | .13 | .049 | [-0.04,0.71] |  |
| T1 HairE ^b^ |  | .12 | .088 | [-1.26, 12.27] |  |
| *Note. β =* Standardized beta coefficient. Bca CI = 95% bias corrected and accelerated bootstrap confidence interval (2, 000 iterations), adj. *R^2^.* = Adjusted coefficient of determination. ASQ_personal_ = Personal social scale of the Ages and Stages Questionnaire– 3. EPDS = Prenatal depressive symptoms measured by the Edinburgh Postnatal Depression Scale. HairE = hair cortisone concentrations.  ^a^ Assessed at T1 DREAM (*M* = 26.78 pregnancy week, *SD* = 5.63). ^b^ Assessed at T1 DREAM_HAIR-BABY_ (*M* = 10.30 days after birth, *SD* = 4.08).  Model 1: *F*(1, 178) = 2.35, *p* = .127  Model 2: Δ*F*(1,177) = 2.44, *p* = .120, Δ*R*^2^= .01 | | | | | |
